# A Theoretical and Practical Treatise on Wounds from Weapons

**Published:** 1835-01-01

**Authors:** 


*Traité Théorique et Pratique des Blessures par Armes de Guerre, redigé d'après les Leçons Cliniques de* M. le Baron DUPUYTREN, Chirurgien en chef de l'Hôtel Dieu, *et publié sous sa direction* par MM. les Docteurs A. PAILLARD et MARX. *Tome Second.* —Paris et Londres, 1834.

*A Theoretical and Practical Treatise on Wounds from Weapons; being the Substance of the Clinical Lectures of the* Baron DUPUYTREN, *published under his direction* by Drs. A. PAILLARD and MARX. *Second Volume.*—8vo. pp. 527.

IN our last Number we presented our readers with an outline of the contents of the first volume of this work, and we then had occasion to speak highly, both of the soundness of its surgical doctrines, and of the eloquence and spirit of its language. Nothing more need be said of the merits of the present volume, than that it is evidently the produce of the same master-hand, and is in every respect worthy of its predecessor.

It opens with a very complete history of hemorrhage, which commences with a caution to young surgeons against being misled as to the quantity of blood lost, either by the representations of attendants, or by a hasty survey of its appearance and colour. The best method of judging of the extent of hemorrhage is by its effect on the constitution, though this will of course be much modified by the rapidity with which the blood flows.

The colourless skin and feeble pulse, the general coldness of the body, and loss of strength; subsequently, the irregularity of the pulse, the alternate failure and acceleration of the expansions of the chest, syncope more or less frequent and prolonged, palpitations of the heart, vomiting, inability to retain either food or drink, and spasmodic or convulsive movements; are the principal symptoms indicative of a great loss of blood, and demanding instant attention.

Copious and repeated hemorrhages have a much more lasting effect than is generally supposed: they occasion for a long time paleness, languor, and weakness, which may last months, years, or even during the whole life, according to the quantity lost, and the age and constitution of the patient. Meanwhile, the blood is of a light colour, and does not readily coagulate, and for a long period the patients retain a chlorotic and bloodless appearance; so difficult is the restoration of its fibrinous and colouring matter. Nevertheless, during this time local congestions frequently occur, the presence of which is shown by restlessness, sleeplessness, spasms, drowsiness, giddiness, and pulsations in different parts of the body, as the head, neck, heart, &c. But these symptoms are easily removed by the abstraction of small quantities of blood. The remedies for this state of system are repose of body and mind, good nourishment, especially of the animal kind, broths, jellies, tonics, steel, and country air: these, continued for a greater or less period, according to circumstances, will generally restore the health to its pristine vigour.

Children are unable to bear large hemorrhages; though, should they survive the first depression, and the convulsions which frequently follow them, the blood is more easily replaced than in adults. Women, on the other hand, especially those in the pregnant state, support the loss of blood better than men; and they likewise possess a greater power of reproducing it.

The quantity of blood which may be removed without occasioning death greatly varies in different persons, and also according to the rapidity with which it flows. Instances have occurred where twenty-five pounds of blood have been lost in a few days; a larger quantity than circulates at any one period in the body. In such cases there is extreme thirst, and the fluid taken into the stomach is immediately absorbed, so that the blood is diluted, and becomes perceptibly of a lighter colour.

We have thus abridged the general remarks upon hemorrhage; not that we have been prompted to it by an idea that these points are unknown to the readers of this Journal, but rather that, by the selection of what is familiar to them, we may show them the comprehensive and practical manner in which our author discusses the various parts of his subject. The restlessness, however, and other symptoms indicative of local congestion, should not be treated with small bleedings, but with opium.

Dupuytren lays great stress upon the peculiar colour (*couleur d'amaranthe*) of the blood when diluted after large hemorrhages, and states that it cannot be successfully imitated by the admixture of any substance with the blood out of the body. We must confess that we have not recognized this colour by the author's description; for, though the venous blood of weak persons is certainly lighter than that of the robust, yet, as far as we know, it only possesses the tint of blood diluted with serum.

The section upon *Arterial Hemorrhage* commences with a description of the difference between arterial and venous blood, followed by an account of the structure of arteries. In speaking of the use of the middle coat, which he believes to be fibrous, he attributes to it the powers both of retraction and contraction. The retractile power is most remarkable in beasts of prey, less so in wild herbivorous animals, and still less in the domesticated, especially in such as are fat. In the human subject it is greater during infancy and youth than in adult life; it decreases in old age, till it ceases altogether, from the ossification of the vessels. Hence arises the extreme difficulty of stopping hemorrhage in young persons, when small vessels are only partially divided; and, on the other hand, the little liability to it in advanced life. The coagulability of the blood varies also in different animals and in man: this tendency exists to the greatest extent in birds, and nearly as powerfully in carnivorous animals, but is much weaker in herbivorous creatures. In man it is more marked than in the latter class, though inferior to the two former. The importance of these remarks is evident, in comparing the results of experiments upon animals with that which takes place in the human subject. The coagulation of blood is more rapid in the negro than in the white, in adult man than in the infant, the old man, or the woman; in a healthy individual, than in the weak; in a sanguine and bilious, than in a lymphatic constitution; in patients with inflammatory diseases, than in those affected with atonic maladies; in such as have never lost blood, than in those in whom it is diluted by repeated hemorrhages. Hence the author cautions the surgeon against satisfying his patient's thirst; for by this means the blood becomes diluted, and loses that coagulating power to which he looks for the suppression of the bleeding.

Our author then proceeds to detail the various methods of arresting arterial hemorrhage; and, not content with relating how they are severally to be employed, he describes with great minuteness the rationale of their action. The editors too, in their notes, do full justice to the labours of our English physiologists, especially of Hunter and Jones.

From this part of the work we shall select the following summary of the author's opinions upon the present state of our knowledge with regard to the torsion of arteries.

After describing the methods of Amussat and Thierry, he thus proceeds:

"Finally, the torsion of the extremities of arteries divided in an operation, or by a wound, has in its favour many different experiments upon animals, besides some successful trials upon the human subject. If it should succeed, it will greatly aid in promoting union by the first intention; for the ligature, acting as a foreign body, irritates the bottom of the wound, and frequently prevents this process. But at present torsion wants the sanction which time and numerous trials alone can bestow on so important an operation. The method of torsion, when it requires the complete division of the artery prior to its application, is neither free from objection, nor is it applicable in all cases. The plan of twisting a whole artery, as proposed by M. Thierry, requires a combination of circumstances which is rarely found, and then it risks the rupture of the vessel, and consequent hemorrhage. Even when it can be employed, it requires uncommon dexterity, and is much more difficult in its application than the ligature. At any rate, however, it must be confessed that the experiments upon which the plan of torsion is founded are highly important; and it is to be hoped that some useful method will spring from them. Meanwhile, prudence demands that we should employ it with great caution, and in such places only where we are sure of finding other assistance against the accidents to which they may give rise when they fail." (P. 52.)

The Baron has entered at considerable length into the subject of *Venous Hemorrhage;* but, as there is but little novelty in his remarks, we shall content ourselves with quoting his directions on the method of applying compression to veins, which, if sufficiently attended to in practice, are not generally laid down so clearly in books.

"Compression, applied badly, instead of being serviceable, is frequently highly injurious to the patient. In order to arrest arterial hemorrhage, pressure should be applied, so that the sides of the artery may be put in contact with one another, and its cavity entirely obliterated; but, when applied to veins, it should only slightly support their parietes; and this is generally sufficient to prevent the further effusion of blood. If, however, the pressure be so great as to obliterate their cavity, as in the arteries, it always occasions a collection of blood beneath the compressed part, which is soon evidenced by the swelling of the limb and the violet colour of the skin. This is soon followed by extravasation of blood, either from the wounded vein, or from those in its neighbourhood. However small may be the size of the vein, the pressure should be confined to the point wounded, and neither be applied above or below; and its object should only be to support, and not to obliterate the vessel." (P. 63.)

The author insists much on the influence of deep inspirations in increasing venous hemorrhage, and he therefore cautions the surgeon to prevent the patient's cries as much as possible. Every one who has witnessed any serious surgical operation must perceive the wisdom of this remark.

While on this subject, our author devotes a section to the consideration of the effect of air entering the veins, and another to varicose aneurism; but we shall pass over them, and proceed to his observations upon *Visceral Abscesses*, or *deposits of pus*. He commences by referring us to many authors who long since noticed the existence of abscesses at a distance from the injured part, though he gives the credit to modern surgeons of having directed attention to them as a frequent cause of death after accidents. Nothing is more obscure or insidious than the symptoms of these internal inflammations and suppurations, after severe wounds or operations. They are, however, generally preceded by traumatic fever, of a graver character than might be expected from the appearance of the wound. This fever continues for a considerable period, with occasional exacerbations, preceded by shiverings, which, occurring from the fifth to the tenth day, are rather to be regarded as the signs of the formation of pus, than of the existence of inflammation. These rigors are the only symptoms that can be depended upon: they make their appearance almost every day, and so regularly, that the patient is not unfrequently supposed to labour under intermittent fever. Very frequently an attack of indigestion takes place at the onset of the disease; the sore becomes unhealthy and pale, and the discharge diminishes in quantity, and becomes of a serous nature. The patients, nevertheless, complain of little or no pain, but suffer only from fever and extreme uneasiness. The utmost caution often fails in discovering the seat of the disease; but, as it proceeds, the fever becomes more severe, the discharge dries upon the surface of the sore, the skin becomes slightly jaundiced, the tongue is dry and black; muttering delirium supervenes, and the respiration becomes affected. It is then only that the symptoms are sometimes recognised as those of suppuration; the patient dies, and the examination of the body verifies the late prognosis, with the exception of the mischief being more extensive than could have been suspected. The progress of the disease will of course be modified, according to the organ attacked. When the brain is affected, there will be more delirium; when the liver, there will be tenderness in the right hypochondrium, vomiting, &c.

To this excellent history of the symptoms of internal suppuration, which we have abridged from the author's account, little can be added with advantage. We think, however, that he has rather over-rated the difficulty of detecting the seat of the malady. As far as our experience goes, we should say that the patient ordinarily complains to his nurse or attendant of darting pains in the part affected, and that, if he do not mention it to his surgeon, it is because the mind participates in the feebleness of his body, and he is unwilling to exert himself to give an account of his feelings, or possibly he may have forgotten them ere the surgeon's next visit. The appearance of the sore, too, is so peculiar as to deserve a more lengthened description; for, whether the matter be confined in the immediate neighbourhood of the wound, or whether these visceral abscesses are forming, the same process takes place. The ulcer first becomes pale, then yellowish streaks appear intersecting it in various directions, and afterwards all the granulations become absorbed. The surface is smooth and glossy, with the exception of a few pale reddish points, which seem like vain attempts at granulation. The rapidity with which this change takes place is quite astonishing: a sore may be healthy in the morning, but in the evening it may be in the last described state.

Another circumstance that may be mentioned in connexion with these abscesses, is the length of time that the disease may exist before it terminates fatally. We have ourselves seen a patient live seven weeks after the first rigor in this hopeless state, and they very commonly survive three or four weeks. There is, however, a fortunate provision in these cases to mitigate their miserable condition, viz. an utter unconsciousness of their extreme danger, so that they will constantly answer the surgeon's interrogatories by assurances of being much better, while they are really within a few days of death.

The morbid appearances depend upon the length of time which the patient survives after the first onset of the disease. There may be either simple congestions of the vessels, a small ecchymosis, an infiltration of serum, pus either inclosed in one of the great serous cavities, or diffused through the cellular membrane; and, lastly, circumscribed abscesses in the parenchyma of some viscus. These abscesses may also occur in the limbs, the calf of the leg, &c.

The question now arises, how are these abscesses to be accounted for? The most common theory is, that of the absorption of pus from the surface of the wound: there are others who believe that they are occasioned by inflammation of the inner surface of the veins. But the author objects alike to both of these hypotheses, and offers the following explanation of the method of their formation. The traumatic fever which accompanies wounds at the period of their inflammation is intended to form a certain quantity of pus; it is (if we may be allowed the expression) a pus-forming fever, *(fièvre pyogenique;)* that is to say, it gives to the fluids that flow towards the affected part such a nature as is requisite for their conversion into pus. Is it then to be wondered at that this disposition to form pus extends beyond the fluids going to the inflamed part, and that, in consequence of this disposition becoming more general, suppurations should take place in those parts which might be in a somewhat disordered state prior to the accident or operation. There is no doubt that the existence of suppuration in one part of the body inclines other parts to take on the same process, or, in other words, suppuration produces suppuration in any part where a previous tendency may have existed.

We shall not enter into any examination of the truth of this hypothesis: it appears to us as good as any other, and in one respect better; for, if this be true, it will lead surgeons to pay still greater attention to the minutiæ of the patient's constitution, previously to undertaking any important operation; whereas, if the theory of its absorption by the veins be correct, we are utterly ignorant of the circumstances favouring this absorption, and therefore can do nothing to prevent its occurrence. There is one circumstance that will support our author's views which he has not mentioned, viz. that these abscesses never, or at least very seldom, occur in private practice, where greater care is taken about exposing the patients to draughts of air, and other exciting causes of fever, than can be bestowed in large public institutions.

The observations on *Hospital Gangrene* are extremely good, but, as far as we can judge, in nowise original. Our author generally employs the nitrate of mercury as the local dressing; in this country we are in the habit of using nitric acid, or the liquor arsenicalis, as recommended by Blackader. We have ourselves seen considerable benefit arise from the use of the Barbadoes tar, spread thickly upon lint, and laid upon the sore twice a day.

In the chapter upon the *Diseases to which Cicatrices are liable*, the Baron gives excellent directions how to detect people in voluntarily cutting off their own fingers; but, as we are not very partial to this amusement on this side of the water, our readers will forgive us for passing over them, to make room for some of his remarks on *Wounds of the Head*.

In the section upon punctured wounds of the scalp, our author enters into the subject of diffuse inflammation of the cellular tissue under the aponeurosis of the occipito-frontalis muscle, of which he gives a good description. There is one point only on which we should differ with him, and that is in the treatment. He recommends a free division of the parts as a preventive measure, and to this we have no objection; but he goes on to add, that recourse should be had to large bleedings from the arm. Now, from this practice we entirely dissent; for, as far as our experience goes, we should say that, under ordinary circumstances, bleeding is one of the most efficacious means of exciting diffuse inflammation. In cases of injury of the head where mischief has been done within the cranium, it may be requisite to bleed, and to run our chance of the inflammation externally: nay more, in certain very full-blooded and robust persons, a small venesection may be advantageous. But in such constitutions as are generally met with in large towns, (and it is in such cases that the disease is most likely to be severe,) bleeding ought never to be employed. Diffuse inflammation is a disease of weakness, for, where the patient is strong, the inflammation is circumscribed; the parts are endowed with but little vitality; and, if this be diminished by loss of blood, they readily fall a prey to the progress of the malady. This opinion is not theoretical; we can refer to a large number of cases, in which it would be shown that those patients with wounds of the scalp who were bled, almost always were attacked by diffuse inflammation.

We are, however, but little inclined to argue with such a man as Dupuytren, for we feel that we are more fitted to learn from him; and, as our readers are probably of the same sentiments, we thall abridge some of his most practical observations upon severe injuries of the head, without discussing his opinions.

Lesions of the cranium from pointed instruments may remain without doing mischief for a number of years. The following case illustrates this fact, and at the same time shows how far good surgery may go in saving the life of a patient.

"Eight or ten years ago, a young man received a stab on the head, and the knife broke in the bone. This was not detected at the time; the edges of the wound were brought together, and united. He remained perfectly well for several years, excepting occasional pains in the cicatrix. At the end of this time, without any known cause, he was attacked with fever and stupor; and, on examining the cicatrix, a foreign body was felt protruding beneath it. A trepan was applied, and a piece of the blade removed. The bad symptoms continued, and the opposite side of the body became paralysed. Dupuytren divided the dura mater, but nothing escaped; he plunged a bistoury into the brain itself, a large quantity of matter spirted out; before evening the fever, delirium, and coma had subsided, and the patient ultimately recovered." (P. 146.)

Where a portion of the skull has been cut away by a sabre, the patients frequently do well; the surgeon, however, should be extremely cautious to remove the smallest fragments of bone that may be loose. The Baron cautions us against trusting to the hope that, because the fragments are attached to soft parts, they may still be saved, as such an expectation always proves fallacious. Every fragment of bone should be removed, and the flaps of integuments laid down, in order to obtain as much union by the first intention as possible.

Our author's opinions upon the subject of *Trepanning* are extremely sound: he is neither a partizan of the Pott school, who would trephine every fissure of the cranium, nor, on the other hand, does he run into the opposite extreme of leaving too much to the restorative powers of nature. The fact of there being fracture, with depression of the bone, is not, in his judgment, sufficient to justify the operation, unless there are also symptoms of compression of the brain. He relates a case of a banker in Paris, with fracture of the skull, and depression of a portion of the frontal bone. As no symptoms of compression were present, the parts were left tranquil, and the gentleman recovered without any ill consequences manifesting themselves, and he is now as able to conduct his affairs as before the accident, though the bone remains depressed. In cases where it is difficult to distinguish the situation of the coagulum pressing on the brain, as, for instance, when the fracture is occasioned by the *contrecoup*, our author would still advise the use of the trephine, leaving it to the judgment of the surgeon to determine on the spot to which it should be applied. His reasons for so doing are, that we can make the case no worse, and we may perhaps do good. We are willing to give all due force to this argument, though we doubt if we can follow him to his next application of it. "For," says the Baron, "in certain cases of deep extravasation, we are obliged to cut through the dura mater, the arachnoid, and even the brain itself, if the coagulum be near the surface;" and, by way of justification of such bold measures, he adds, "that Boyer did not scruple to plunge his bistoury deep into the brain." (P. 183.)

Now, for our own parts, we do not know how it is to be decided that the coagulum is near the surface; and, should an English surgeon plunge his bistoury deep into the brain, and fail in discovering the coagulum, we apprehend that the coroner would probably interfere to give his medico-legal opinion on the point.

The chapters on *Wounds of the Face* are replete with sound surgery, but there is little in them that is either novel or interesting.

The *Wounds of the Orbit and Eye, of the Nose, Ear, Cheeks, and Jaws,* form separate sections, but their treatment is nearly similar, and may be comprised in a few directions, viz. to clean the wounds, and extract all foreign substances; and, as soon as the sloughs have separated, and the inflammation diminished, to draw the edges together, and keep them as nearly as possible in contact with one another.

We have said that there is but little novelty in our author's observations on wounds of the face; but we must make an exception of the following brilliant operation.

"Charles Antony Mercier, ætat. thirty-six, a soldier in a dragoon regiment, attempted suicide in 1830; but, fearful lest his courage should fail, he drank a large quantity of spirits, to prime him for his attempt. Having loaded his pistol with two balls, he placed the point of it under the jaws; but, being completely intoxicated, his hand shook, and the explosion broke the lower jaw to pieces, and carried away a large quantity of the surrounding soft parts. The surgeon who saw him in this state merely applied some simple dressings, under which the wound healed, leaving however a dreadful deformity. The portion of the lower jaw between the canine tooth on the right side, to the ascending ramus of the left side of the jaw, was totally destroyed; and nearly all the lower lip, together with the integuments covering the chin, so far back as the os hyoides, had also been shot off. Thus the lower part of the face presented one great cavity, of which the superior maxillary bone formed the roof, and the os hyoides the floor, over which the saliva was constantly trickling. The masseter and internal pterygoid muscles, having no antagonists, had raised the portion of the jaw upwards nearly to the nose, and projecting before the canine tooth.

"The only question to be considered by the operator was, how far this portion of the jaw could be saved for the purposes of mastication. Dupuytren decided on its removal, as it could not, from its situation, be in the least serviceable without the division of the masseter and pterygoid muscles; and then it would be useless from want of power to move it. The operation was performed as follows:

"A transverse incision was made, about one inch and a half in length, in the right cheek, at the commissure of the lip, and the portion of the jaw dissected, and removed as far as the second molar tooth by the chain-saw. The other edges of the opening were then pared off, and the parts drawn forcibly together by sutures in a direction from above downwards. The sides of the wound were in contact, excepting a small portion at the lower parts, where they were too firm to admit of extension. Bandages and graduated compresses were then applied, to assist the sutures in preserving the situation of the parts. A large portion of the wound united by the first intention, and the rest, with the aid of the nitrate of silver, subsequently filled up with granulations, so that the face regained nearly its natural appearance." (P. 267.)

We have greatly abridged the account of this operation, (which is from the pen of M. Hippolyte Larrey, the son of the celebrated surgeon;) but even our account of it, which does not enter into minute details, requires no comment to convince our readers that none but a first-rate anatomist, and a most dexterous operator, could have succeeded in so arduous an undertaking.

The chapter on *Wounds of the Neck* is full of excellent cases; as also that on *Wounds of the Chest*. In the latter are detailed several instances of severe wounds of the heart that did not prove fatal for several days; one especially, where life was prolonged for twenty days, though the left ventricle was transfixed by a watchspring. Such instances, though highly curious, have but little practical utility, as no case has yet been recorded of complete recovery after an incised wound of the heart. We shall terminate by an abridgment of our author's observations on *Gunshot Wounds of the Abdomen*.

Many instances, of course, occur, in which such mischief is done to the contents of the abdomen as to cause immediate death, or, if the patient survive long enough to admit of receiving surgical aid, all that can be done is to stop the hemorrhage; and, having dressed the wound with some simple dressing, to endeavour to prolong life for a few hours, by the exhibition of stimulants. Another set of cases which escape instant death, end fatally in a few days, from the inflammation consequent on the injury. Here, however, the surgeon's province is more extensive: he must return the protruded viscera into the cavity of the abdomen; remove, if it can be easily done, all extraneous substances; prevent the occurrence of inflammation by all the means in his power, and give issue to any effusion that may take place afterwards.

When the stomach is wounded, the injury is generally fatal. Inflammation sometimes, however, arises; lymph is effused which unites its serous coat with the peritoneum lining the walls of the abdomen, and thus extravasation of its contents being prevented, a perfect cure is obtained by means of antiphlogistic treatment, rest, and total abstinence. No attempt should be made to extract the ball.

Wounds of the small intestines are generally mortal, as several of the folds are most probably injured. If, however, the wounded portion of intestine can be seen, it should be drawn towards the parietes, and be secured there by a suture. Nature will sometimes of herself effect a cure; adhesions take place around the edge of the wound, and prevent effusion of its contents. When however this effusion occurs, severe peritonitis is excited, and the surgeon's art is of no avail. Violent pain, colic, and vomiting, with smallness and feebleness of the pulse, are the symptoms indicative of approaching dissolution.

When the cœcum and colon are the injured parts, a more favourable result may be anticipated. As they are not covered entirely by peritoneum, the effusion of fæces may take place externally, and after an attack of inflammation, more or less severe, the patient is left with an artificial anus. Great care should be taken in removing any bridles, or cleansing the edges with the knife, lest the peritoneum be wounded, which would render the prognosis much more unfavorable.

Wounds of the rectum are generally complicated with wounds of the bladder or sacrum. When the bladder is wounded, our author advises the free division of the external sphincter, in order that the fæces may have a free escape from the rectum, as is practised in this country for fistulous communication between these viscera.

Injuries of the liver are for the most part fatal in a few days, either from hemorrhage or inflammation. The whole treatment must consist of bleeding, abstinence, and effervescing draughts, to moderate the vomiting. If the ball be near the surface, and there is no hemorrhage, an attempt may be made to extract it. Under this treatment a few cases have recovered.

Gunshot wounds of the spleen are always mortal.

Dupuytren has seen only one case in which the kidney has been wounded by a bullet. In this instance urine escaped from the wound; the ball was not extracted, but the patient recovered in fourteen days. It would be expected that, if thc urine did not flow through the external wound, that it would make its way either into the cellular substance in its neighbourhood, or into the cavity of the peritoneum; in either of which cases death would be certain. Wounds of the bladder are generally followed by extravasation of urine, the symptoms and treatment of which should be known to every one. A few cases are recorded where the bullet remained in the bladder, and produced all the symptoms of calculus, for which the operation of lithotomy is the only remedy.

We have now brought to a conclusion our notice of this work, the best and most comprehensive upon the subject that has yet fallen into our hands. The author has been long recognised as the first surgeon in Europe; the perusal of these lectures will lead us to doubt if he be not also one of the best speakers. For the pleasure and improvement that we have derived from these volumes we tender our thanks to the editors, who, by thus connecting their names with that of Dupuytren, will earn a lasting reputation.

